# Effect of intratracheal dexmedetomidine combined with ropivacaine on postoperative sore throat: a prospective randomised double-blinded controlled trial

**DOI:** 10.1186/s12871-022-01694-6

**Published:** 2022-05-14

**Authors:** Jingyi Niu, Rui Hu, Na Yang, Yan He, Hao Sun, Rende Ning, Junma Yu

**Affiliations:** 1 Department of Anaesthesiology, The Third Affiliated Hospital of Anhui Medical University, The First People’s Hospital of Hefei), 390 Huaihe Road, Hefei, 230061 Anhui China; 2 Wannan Medical College, 22 West Wenchang Road, Wuhu, 241002 Anhui China; 3 Department of Orthopaedics, The Third Affiliated Hospital of Anhui Medical University, The First People’s Hospital of Hefei), 390 Huaihe Road, Hefei, 230061 Anhui China

**Keywords:** Dexmedetomidine, Ropivacaine, Postoperative sore throat, Endotracheal intubation, General anaesthesia

## Abstract

**Background:**

The present study aimed to investigate whether intratracheal dexmedetomidine combined with ropivacaine reduces the severity and incidence of postoperative sore throat after tracheal intubation under general anaesthesia.

**Methods:**

Two hundred patients with American Society of Anaesthesiologists physical status I-II who were subjected to general anaesthesia were randomly divided into four groups, namely, Group D, Group R, Group DR and Group S; these groups received intratracheal dexmedetomidine (1 µg/kg), 0.8% ropivacaine (40 mg), dexmedetomidine (1 µg/kg) combined with 0.8% ropivacaine (40 mg) and normal saline before endotracheal intubation, respectively. The primary outcomes were the incidence and grade of sore throat and hoarseness at 2 h and 24 h after surgery. Moreover, the modified Observer's Assessment of Alertness/Sedation Scale results were recorded at each time point. The secondary outcomes were intraoperative haemodynamic fluctuations, intraoperative anaesthetic drug requirements, and adverse reactions during and after surgery. The patients’ vital signs before induction, before superficial anaesthesia, after superficial anaesthesia, before intubation, after intubation, and 1 min after intubation were recorded. The use of anaesthetic drugs and occurrence of adverse effects were also recorded.

**Results:**

The incidence and severity of sore throat were significantly lower in Group DR than in the other three groups 2 h after the operation, but they were only significantly lower in Group DR than in the control group 24 h after the operation. Moreover, compared with Group S and Group D, Group DR exhibited more stable haemodynamics during intubation. The doses of remifentanil and propofol were significantly lower in Group DR than in the other groups.

**Conclusion:**

The combined use of dexmedetomidine and ropivacaine for surface anaesthesia before intubation significantly reduced the incidence and severity of postoperative sore throat. This treatment also decreased anaesthetic drug requirements and intraoperative haemodynamic fluctuations and caused no adverse effects.

**Trial registration:**

This clinical research was registered at the Chinese Clinical Trial Registry (ChiCTR1900022907, Registration date 30/04/2019).

## Background

Endotracheal intubation is a common procedure used during spinal surgery under general anaesthesia that provides effective mechanical ventilation. However, endotracheal intubation is the prominent cause of airway mucosal injury, which may result in postoperative sore throat (POST) [1]. Even though POST is a minor adverse event during anaesthesia recovery [2], it causes discomfort for the patient and reduces patient satisfaction with anaesthesia. POST was recently reported to have an incidence of up to 62% after general anaesthesia [3]. The incidence of POST is correlated with age, sex, tracheal tube size, endotracheal tube cuff pressure and other factors [4]. Various methods have been applied to reduce the incidence and severity of POST, including IV administration of lidocaine or dexamethasone [5, 6]; replacement of a double-lumen tube with an endobronchial blocker [7]; and prophylactic use of nebulised ketamine, magnesium, or corticosteroids [8, 9].

Dexmedetomidine is a highly selective adrenergic α-2 receptor agonist that is used for sedation, analgesia, and anxiety inhibition [10]. Ropivacaine is a sodium channel blocker that can affect sensory-motor block. Ropivacaine exhibits good liposolubility and long-acting effects and reaches an acceptable blood concentration via the mucous membrane [11]. Topical ropivacaine anaesthesia can effectively reduce haemodynamic responses during intubation and extubation and reduce the incidence of postoperative throat pain and cough [12]. It was reported that intracheal dexmedetomidine administration 30 min before the end of surgery leads to a smooth extubation and balanced anaesthesia recovery [13]. Several studies have shown that the combination of dexmedetomidine with ropivacaine effectively improves the efficacy of analgesia and extends the duration of analgesia after surgery [14–16]. Therefore, we hypothesised that intratracheal dexmedetomidine combined with ropivacaine would decrease the incidence and severity of POST after tracheal intubation.

## Methods

This study was a prospective, patient- and investigator-blinded, controlled clinical trial with equal randomization, and it was approved by the Ethics Committee of the Third Affiliated Hospital of Anhui Medical University (No. PJ2019–03–01, approval on 26/03/2019) and registered in the Chinese Clinical Trial Registry (www.chictr.org.cn, ChiCTR1900022907, Registration date 30/04/2019). The study took place at the Third Affiliated Hospital of Anhui Medical University, The First People’s Hospital of Hefei. Each patient provided written informed consent before participation in the study. All the methods were carried out in accordance with the Declaration of Helsinki.

American Society of Anaesthesiologists (ASA) I-II, airway Mallampati class I-II, 20- to 65-year-old patients scheduled for spinal surgery with general anaesthesia were recruited. The exclusion criteria were as follows: ASA ≥ III; body mass index (BMI) ≥ 30 kg·m^−2^; severe cardiovascular, liver, and kidney dysfunction; uncorrected vision or hearing impairment; difficult airway or history of maxillofacial and neck surgery; chronic use of opioids or sedatives; chronic respiratory disease; recent respiratory tract infection; chronic cough; anaesthesia time more than 4 h; time without extubation more than 1 h; and transfer to the intensive care unit while still intubated.

Subjects were randomised to Group D, Group R, Group DR or Group S with an allocation ratio of 1:1:1:1 according to a computer-generated random number table. The details of the group assignments were sealed in sequentially numbered opaque envelopes. The anaesthesia nurses were given the sealed envelopes and asked to prepare the experimental drugs in syringes according to the group assignments; there was no difference in appearance among the groups. All the patients and anaesthesiologists were blinded to group assignments. An anaesthesiologist administered the drugs to the patient according to the coding number after anaesthetic induction. Another anaesthesiologist recorded intraoperative data and interviewed the patient about sore throat and hoarseness after the surgery.

**Fig. 1:**
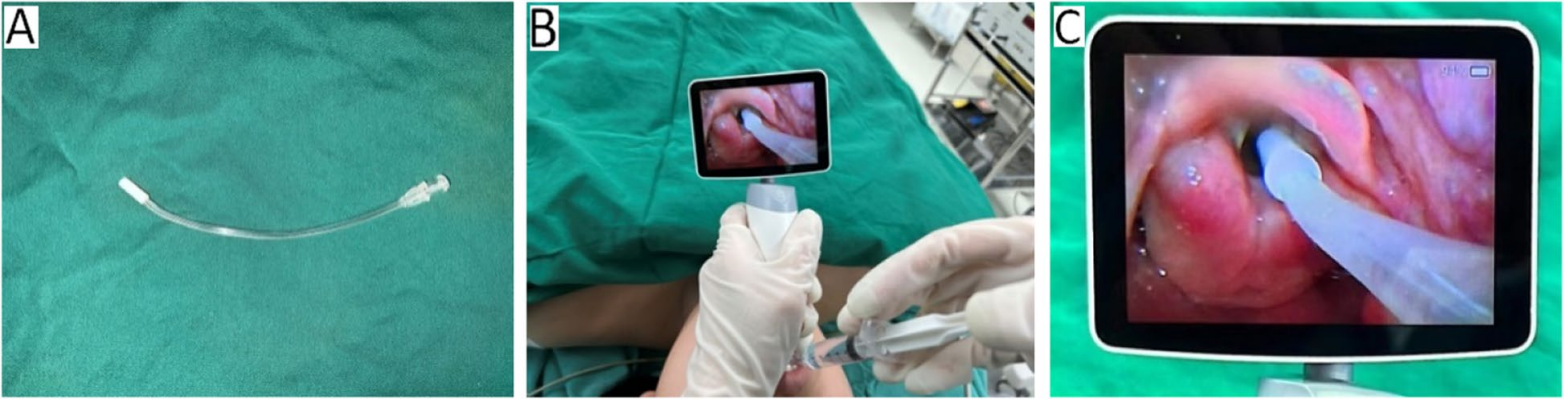
A disposable laryngo-tracheal mucosal atomization device (**A**); Five ml of the treatment drug was sprayed onto the tracheal mucosa and glottis to achieve uniform surface anaesthesia (**B** and **C**)

Electrocardiography (ECG), blood oxygen saturation (SpO_2_), heart rate (HR), invasive blood pressure (IBP), and respiratory rate (RR) were monitored after the patients were admitted to the operating room. IBP and HR were recorded as the basal blood pressure and basal heart rate after 10 min of resting. The vein channel was established with an 18-gauge indwelling needle. Anaesthesia induction was performed by a senior anaesthesiologist. No subjects received preoperative medication. Oxygen was inhaled via a mask for 3 min, and all the patients received 0.3 μg/kg sufentanil, 1.5–2.5 mg/kg propofol and 0.2 mg/kg cisatracurium besilate via intravenous administration for induction. After the induction of general anaesthesia for 3 min, we used a disposable laryngo-tracheal mucosal atomization device (TUORen Medical Equipment Co., Henan, China; Fig. 1 A) to spray 5 ml of the treatment drug onto the tracheal mucosa and glottis to achieve uniform surface anaesthesia (Fig. 1 B and C). The drug treatments (diluted to 5 ml in each group) used in the four groups were as follows: Group D: dexmedetomidine (1 µg/kg); Group R: 0.8% ropivacaine (40 mg); Group DR: dexmedetomidine (1 µg/kg) combined with 0.8% ropivacaine (40 mg); Group S: saline. Next, endotracheal intubation was performed by two senior anaesthesiologists using a video laryngoscope after 2 min of assisted breathing, and we got successful intubation within 30 s at first time in all patients. The internal diameter of the endotracheal tube used was 6.5 mm for female patients and 7.0 mm for male patients. After successful intubation, mechanical ventilation was resumed, and the end-tidal carbon dioxide pressure was controlled at 35–45 mmHg.

Anaesthesia was maintained by 1% sevoflurane, 3–12 mg·kg^−1^ h^−1^ propofol, 0.1–0.3 μg·kg^−1^ min^−1^ remifentanil, and intravenous cisatracurium besilate intermittently to maintain a suitable depth of anaesthesia (bispectral index, BIS 40–60). Cuff pressure was maintained at 20–25 mmHg with the continuous monitoring of a pressure transducer [17]. Ondansetron (4 mg) was administered before the end of surgery. When anaesthesia was discontinued and the patients were restored to spontaneous respiration, 1 mg of neostigmine and 0.5 mg of atropine were administered to antagonise the muscle relaxants. The tracheal tube was removed when the patients were conscious, the RR was more than 12 breaths/min, the tidal volume was more than 6 ml/kg, there was swallowing and cough reflex recovery, and the SpO2 was ≥ 95%.

The primary outcome was the incidence and grade of sore throat and hoarseness at 2 h and 24 h after surgery, and the secondary outcome was intraoperative haemodynamic fluctuations, intraoperative anaesthetic drug requirements, and adverse reactions during and after surgery. The anaesthesia nurse recorded patient characteristics and assessed the ASA statuses, BMI values and Wilson risk sum scores of the patients on the day before the surgery. During the operation, the anaesthesiologist recorded the start time of anaesthesia, time of intubation, start time of the operation, end time of the operation, end time of anaesthesia and time of extubation. The patients’ systolic blood pressure (SBP), diastolic blood pressure (DBP), and HR before induction (T0), before superficial anaesthesia (T1), after superficial anaesthesia (T2), before intubation (T3), after intubation (T4), and 1 min after intubation (T5) were recorded. Moreover, the use of anaesthetic drugs (sufentanil, remifentanil, propofol, or cisatracurium besilate) and occurrence of adverse reactions during surgery and after surgery were recorded. The grading of sore throat was as follows [18, 19]: rating of 0, no sore throat; level 1, mild sore throat (complained of sore throat only when asked); level 2, moderate sore throat (self-reported sore throat); and level 3, severe sore throat (pain and discomfort in the pharynx that cause hoarseness or vocal change). Grade of hoarseness [19]: rating of 0, no hoarseness; level 1, mild hoarseness (complained of hoarseness only when asked); level 2, moderate hoarseness (self-reported hoarseness); level 3, severe hoarseness (change in voice was observed). The OAA/S score (0–5) was assessed as follows [20]: rating of 0, no response to squeezing of the trapezius; level 1, no response to mild pushing and shaking; level 2, response only to mild shoulder or head shaking; level 3, response only to loud or repeated name calling; level 4, indifference to normal intonation of names; and level 5, quick response to normal intonation. HR < 50 bpm was considered bradycardia, and an OAA/S score ≤ level 4 was considered an adverse reaction.

All the statistical analyses were performed using SPSS version 20.0. The one-sample Kolmogorov–Smirnov test was used to assess the normality of the quantitative data. Quantitative variables are presented as the mean ± standard deviation (SD) or mean ± standard error of the mean (SEM), and categorical variables are presented as numbers (n/%). Quantitative variables with normally distributed data were analysed using one-way ANOVA followed by the Bonferroni post hoc test. Categorical variables were assessed using χ2 or Fisher's exact test. Repeated measures analyses of variance (ANOVAs) were conducted to analyse differences in SBP, DBP and HR at different time points. Four separate two-way repeated-measures ANOVAs were conducted to identify within-subject effects of different groups. *P* < 0.05 was considered statistically significant.

In a pilot study, it was found that the incidence of sore throat 2 h after thoracolumbar spinal operation in 10 patients was 50%. In addition, we found that the incidence of sore throat 2 h after thoracolumbar spinal operation in patients who received laryngo-tracheal dexmedetomidine (1 µg/kg) combined with 0.8% ropivacaine (40 mg) spray was 20%. Through a two-sided test with α = 0.05 and β = 0.2, the sample size was 39 in each group, as calculated using G*Power V.3.1.9.4. Considering the 20% rate of patient exclusion, ultimately, a total of 200 ASA status I or II patients were enrolled in this study.

## Results

**Fig. 2:**
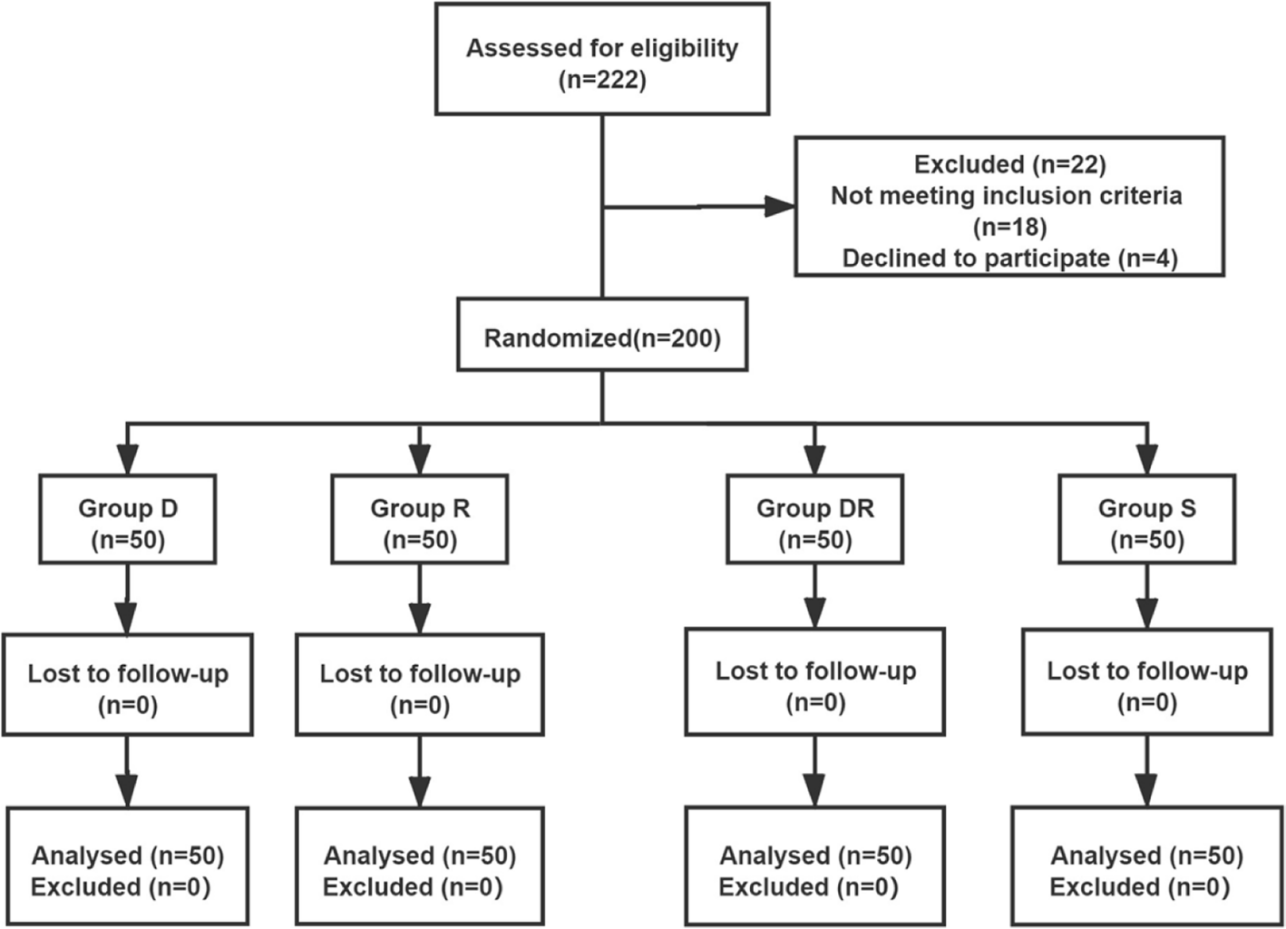
CONSORT flow diagram. *Abbreviations*: Group D: the patients received intratracheal dexmedetomidine (l µg/kg); Group R: the patients received intratracheal 0.8% ropivacaine (40 mg); Group DR: the patients received intratracheal dexmedetomidine (l µg/kg) combined with 0.8% ropivacaine (40 mg); Group S: the patients received intratracheal 5 ml normal saline (all group treatments were diluted to 5 ml)

Two hundred twenty-two patients were assessed for eligibility, and of these patients, 22 were excluded; among these patients, 18 patients did not meet the criteria, and 4 refused to participate in the study. Therefore, in total, 200 patients were randomly assigned to Group D, Group R, Group DR and Group S. None of the patients were lost to follow-up (Fig. 2). There were no significant differences in patient characteristics, duration of surgery, duration of anaesthesia, retention time of the tracheal catheter or extubation time between the four groups (Table 1).

**Table 1:** Demographic characteristics of patients undergoing thoracolumbar spinal surgery

	Group S (*n* = 50)	Group D (*n* = 50)	Group R (*n* = 50)	Group DR (*n* = 50)
Age (years)	50.6 ± 9.5	49.3 ± 10.4	49.0 ± 9.7	50.4 ± 10.4
Weight (kg)	64.8 ± 11.0	63.0 ± 8.9	65.7 ± 10.1	65.8 ± 9.6
Height (cm)	164.2 ± 8.6	164.2 ± 7.2	165.0 ± 6.6	164.4 ± 7.7
BMI (kg/m^2^)	23.9 ± 2.6	23.3 ± 2.3	24.1 ± 2.9	24.3 ± 2.3
Sex (female/male)	27/23	29/21	28/22	30/20
ASA physical status (Ι/Π)	13/37	14/36	16/34	12/38
Wilson risk sum score	1.60 ± 0.67	1.56 ± 0.68	1.48 ± 0.54	1.52 ± 0.68
Duration of surgery (min)	149.3 ± 35.4	157.1 ± 38.1	147.8 ± 33.4	157.8 ± 32.4
Duration of anaesthesia (min)	178.4 ± 35.2	186.8 ± 38.1	178.8 ± 34.7	187.4 ± 31.1
Retention time of tracheal catheter	187.7 ± 34.2	194.0 ± 37.0	184.8 ± 33.7	193.6 ± 30.5
Extubation time (min)	9.6 ± 5.0	9.2 ± 5.3	8.9 ± 4.6	8.6 ± 5.2

Data are presented as the mean ± SD or the number of patients. Group *S* Saline, *D* Dexmedetomidine, *R* Ropivacaine, *DR* Ropivacaine combined with dexmedetomidine

**Fig. 3:**
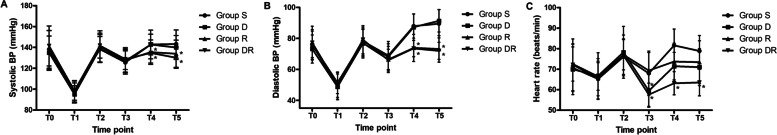
Haemodynamics data are presented as the mean ± standard deviation (SD) or standard error of the mean (SEM). Time point: before induction (T0), before superficial anaesthesia (T1), after superficial anaesthesia (T2), before intubation (T3), after intubation (T4), and 1 min after intubation (T5). Compared with Group S and Group D, Group R and Group DR had lower SBP and DBP at T4 and T5 (**A**: SBP: T4*P = 0.001 and T4*P = 0.002, respectively; T4*P < 0.001 and T4*P = 0.001, respectively, SBP: T5*P < 0.001 and T5*P = 0.015, respectively; T5*P < 0.001 and T5*P < 0.001, respectively. **B**: DBP: T4*P < 0.001 and T4*P < 0.001, respectively; T4*P < 0.001 and T4*P < 0.001, respectively; DBP: T5*P < 0.001 and T5*P < 0.001, respectively; T5*P < 0.001 and T5*P < 0.001, respectively). Compared with Group S and Group R, Group D and Group DR had a lower HR at T3 (**C**: HR: T3*P < 0.001 and T3*P < 0.001, respectively; T3*P < 0.001 and T3*P < 0.001, respectively). Moreover, the HR in Group DR was lower than that in the other groups at T4 and T5 (**C**: HR: T4*P < 0.001, T4*P < 0.001, T4*P < 0.001, respectively; T5*P < 0.001, T5*P < 0.001, T5*P < 0.001, respectively)

The incidence and severity of sore throat was significantly lower in Group DR than in Group S, Group D and Group R (16% vs. 44%, *P* = 0.038, and 40%, *P* = 0.008 and 34%, *P* = 0.002, respectively) 2 h after the operation, but it was only significantly lower in Group DR than in Group S 24 h after the operation (10% vs. 28%, *P* = 0.022). In addition, no differences were detected in the grade of hoarseness (Table 2). Regarding the use of anaesthetic drugs, the doses of remifentanil and propofol were significantly lower in Group DR than in the other groups (*P* < 0.001). The doses of remifentanil and propofol were significantly lower in Group D than in Group S and Group R (*P* < 0.05 vs. Group R, *P* < 0.01 vs. Group S) (Table 3). Compared with Group S and Group D, Group R and Group DR had lower SBP and DBP at T4 and T5 (Fig. 3. A-B), while there was no statistically significant difference at other time points. Compared with Group S and Group R, Group D and Group DR had a lower HR at T3. Moreover, the HR in Group DR was lower than that in the other groups at T4 and T5 (Fig. 3. C). There were no complications associated with the treatment administration in any group during or after surgery.

**Table 2:** Incidence and severity of sore throat and hoarseness in the first 24 h after thoracolumbar spinal surgery (%)

	Group S (*n* = 50)	Group D (*n* = 50)	Group R (*n* = 50)	Group DR (*n* = 50)
Sore throat (0/1/2/3)				
2 h	44 (28/10/10/2)	40(30/11/8/1)	34(33/10/6/1)	16(42/8/0/0) *#
24 h	28 (36/10/4/0)	22(39/8/3/0)	14(43/5/2/0)	10(45/5/0/0) †
Hoarseness (0/1/2/3)				
2 h	12(44/4/2/0)	10(45/3/2/0)	10(45/3/2/0)	4(48/2/0/0)
24 h	4(48/1/1/0)	2(49/1/0/0)	0(50/0/0/0)	0(50/0/0/0)

The incidence and severity of sore throat was significantly lower in Group DR than in Group S, Group D and Group R (*P < 0.05 versus Group S and #*P* < 0.01 versus Group D and Group R) at 2 h after the operation, but it was only significantly lower in Group DR than in Group S at 24 h after the operation (†*P* < 0.05)

**Table 3:** The total doses of anaesthetic drugs

	Group S (*n* = 50)	Group D (*n* = 50)	Group R (*n* = 50)	Group DR (*n* = 50)
Sufentanil (µg)	32.6 ± 5.4	31.3 ± 4.6	32.8 ± 5.0	33.0 ± 4.6
Remifentanil (µg)	1000.5 ± 303.4	859.8 ± 259.5#†	979.9 ± 246.6	586.6 ± 202.5*
Propofol (mg)	854.7 ± 248.1	740.0 ± 211.7#†	838.6 ± 202.8	524.1 ± 165.6*
Cisatracurium besilate (mg)	28.2 ± 4.5	28.9 ± 4.4	27.7 ± 3.9	29.6 ± 3.7

Data are presented as the mean ± SD. The doses of remifentanil and propofol were significantly lower in Group DR than in the other groups (*P < 0.001, respectively). The doses of remifentanil were significantly lower in Group D than in Group S and Group R (#*P* = 0.006 and †*P* = 0.020, respectively). The doses of propofol were significantly lower in Group D than in Group S and Group R (#*P* = 0.007 and †*P* = 0.019, respectively)

## Discussion

The findings of this study showed that intratracheal administration of dexmedetomidine combined with ropivacaine before intubation significantly reduced the incidence and severity of POST. This treatment also decreased anaesthetic drug requirements and intraoperative haemodynamic fluctuations and caused no adverse effects.

POST is a common complication of tracheal intubation under general anaesthesia. However, the exact cause of POST remains unclear. There are two main sources of sore throat: pain from supraglottic structures, possibly caused by direct laryngoscopy, and pain from infraglottic structures, presumably caused by the endotracheal tube or cuff [21]. Puyoet and colleagues further showed that patients with sore throat release mitochondrial DNA into the upper respiratory system and that their neutrophils are stimulated to release mediators associated with pain in a TLR9- and DNAse-dependent manner [22].

In this study, the incidence of sore throat was significantly lower in Group DR than in the other three groups at 2 h and was lower in Group DR than in the control group at 24 h after surgery. The reason may be that as an adjuvant for local anaesthetics, dexmedetomidine extended the duration of ropivacaine efficacy and improved the efficacy of analgesia after surgery. The possible mechanisms are as follows: dexmedetomidine is a highly selective and specific adrenergic α-2 receptor agonist that can be directly applied to the peripheral nervous system, inhibiting C-fibres and A α-fibres [23]; the most abundant α-2A area is the locus coeruleus, and dexmedetomidine acts on the locus coeruleus area, inhibiting nociceptive neurotransmission through the posterior horn of the spinal cord and terminating the propagation of pain signals, which leads to analgesia [24]; and dexmedetomidine promotes the release of acetylcholine from spinal interneurons, which increases the synthesis and release of nitric oxide and participates in the regulation of analgesia [25]. However, Brummett’s study reported that the increased duration of analgesia caused by combining dexmedetomidine with local anaesthetic results from inhibition of the hyperpolarization-activated cation current (Ih current) not from 2-adrenoceptor agonism [26]. Moreover, we hypothesised that the results might be related to the anti-inflammatory effect of dexmedetomidine. In rodent models, some research has revealed that dexmedetomidine decreases the expression of inflammatory mediators, microglial activation and nerve apoptosis [27–30]. A recent systematic review and meta-analysis that included 67 studies demonstrated that dexmedetomidine infusion during the perioperative period was associated with lower concentrations of stress hormones (epinephrine, norepinephrine, and cortisol), interleukin (IL)-6, tumour necrosis factor-α, and C-reactive protein after surgery [31]. To some extent, this mechanism is similar to that of dexamethasone in reducing the inflammatory reactions caused by tissue damage [32], but side effects similar to those of dexamethasone, including hyperglycaemia, peptic ulcer and adrenal suppression [33], were not observed in our study.

To eliminate other risk factors for sore throat, we attempted to maintain the cuff pressure at 20–25 cmH_2_O with a pressure transducer to reduce direct trauma to the tracheal mucosa [34]. We also chose a relatively small tracheal tube, which may provide a better view of the tube through the larynx and decrease the damage associated with tube insertion [35]. Furthermore, intubation and extubation were performed by a senior anaesthesiologist, so no injury was caused by the anaesthetic technique.

We observed that intratracheal dexmedetomidine administration can significantly reduce the use of remifentanil and propofol and decrease intraoperative haemodynamic fluctuations, which was consistent with the results of Le et al. [36]. Intratracheal administration of dexmedetomidine, which is a hydrophilic small molecule, is believed to allow its rapid absorption through the bronchial and alveolar capillary network, facilitating its pharmacological effects, such as reducing sympathetic and laryngeal nerve sensitivity and inhibiting the increase in HR and BP during intubation [13]. In addition, it has been demonstrated that dexmedetomidine can prevent remifentanil-induced hyperalgesia and maintain global haemodynamic stability [37]. However, adverse haemodynamic complications of dexmedetomidine included hypotension, bradycardia and even cardiac arrest [38–40]. In the current study, the HR decreased with the application of dexmedetomidine, but only rare episodes of bradycardia requiring atropine were observed. Most likely, because these complications occur more often in elderly patients with cardiac disease or given high dosages of dexmedetomidine intravenously [3], we restricted enrolment to ASA I-II patients, and intratracheal administration resulted in slower effects than intravenous administration. Moreover, sedation scores were measured in the assessments of outcomes. All patients’ OAA/S scores were higher than level 4, and there was no postoperative delay in recovery.

Dexmedetomidine can be administered intravenously, intramuscularly, or intranasally in clinical practice. It has been suggested that intranasal administration is effective, convenient, and well tolerated [41] but has a later onset time [42, 43]. However, the use of dexmedetomidine combined with ropivacaine as an intratracheal spray was first reported.

This study has a number of weaknesses. First, our study was a single-centre investigation of spinal surgery patients; consequently, generalization of these results to other surgical patients should be conducted with caution. Large samples and multicentre clinical verification are essential in the future. Second, all the patients had ASA I-II status and were 20–65 years old, and their basic condition was good. Elderly critical patients were not analysed. Third, the incidence and severity of POST after intravenous dexmedetomidine administration were not assessed in this study. Finally, we merely observed the effect of intratracheal dexmedetomidine combined with ropivacaine within 24 h after surgery, and the long-term outcomes and adverse effects need to be evaluated.

## Conclusion

Intratracheal dexmedetomidine (1 µg/kg) combined with 0.8% ropivacaine (40 mg) before intubation can significantly reduce the incidence and severity of POST within 24 h after surgery, decrease anaesthetic drug requirements and intraoperative haemodynamic fluctuations, and cause no adverse effects.

## Data Availability

The data used to support the findings of this study are available from the corresponding author upon reasonable request.

## Abbreviations

POST: Postoperative sore throat
ASA: American Society of Anaesthesiology
OAA/S: Observer's Assessment of Alertness/Sedation Scale
BMI: Body mass index
ECG: Electrocardiography
SpO_2_: Blood oxygen saturation
HR: Heart rate
IBP: Invasive blood pressure
RR: Respiratory rate
SBP: Systolic blood pressure
DBP: Diastolic blood pressure
